# Deletion of *Bmal1*, a Component of the Molecular Clock, Exacerbates Kidney Damage After Ischemia–Reperfusion by Decreasing *Pparα* Expression

**DOI:** 10.3390/ijms27094091

**Published:** 2026-05-02

**Authors:** Satoshi Kitaura, Taira Wada, Yoshimasa Asano, Shigeki Shimba

**Affiliations:** Department of Health Science, School of Pharmacy, Nihon University, 7-7-1 Narashinodai, Funabashi 274-8555, Chiba, Japanwada.taira@nihon-u.ac.jp (T.W.); asano.yoshimasa@nihon-u.ac.jp (Y.A.)

**Keywords:** BMAL1, PPARα, kidney, circadian rhythm, ischemia–reperfusion injury

## Abstract

Brain and muscle Arnt-like protein 1 (BMAL1) is a transcription factor that forms heterodimers with circadian locomotor output cycles kaput (CLOCK) and drives transcription from E-box elements, thereby regulating the circadian rhythms of gene expression. The kidney expresses numerous rhythmic genes and exhibits circadian physiological function regulation. Circadian rhythm abnormalities, such as sleep disorders and excessive daytime sleepiness, are particularly frequent in patients with chronic kidney disease (CKD). Furthermore, reduced amplitude and phase disruption in clock gene expression rhythms have been reported in mouse CKD models. These results suggest that circadian disruption is associated with renal pathophysiology. However, the role of BMAL1 in the repair process following acute kidney injury (AKI) remains unclear; therefore, this study aimed to elucidate its role in kidney repair following ischemia–reperfusion injury (IRI). We found that the tamoxifen (TAM)-inducible global *Bmal1* knockout (BKO) mouse kidneys exhibited increased lipid accumulation, enhanced fibrosis, and delayed kidney repair post-IRI, and that these abnormalities were associated with reduced *Peroxisome proliferator-activated receptor alpha* (*Pparα*) expression. Furthermore, treatment with a PPARα agonist reduced these abnormalities in BKO mice. Collectively, our findings demonstrate that the BMAL1–PPARα axis promotes post-AKI kidney repair.

## 1. Introduction

In multicellular organisms, a network of circadian clocks regulates the 24 h behavioral and physiological rhythms. The circadian clock system operates through an autoregulatory network of multiple clock genes at the gene expression, translation, and post-translational modification levels [1]. In this system, brain and muscle Arnt-like protein 1 (BMAL1) plays a crucial role as a transcription factor in regulating the circadian rhythm of gene expression [2,3,4,5,6,7]. BMAL1 forms heterodimers with circadian locomotor output cycles kaput (CLOCK), which drives transcription from E-box elements found in the promoters of circadian-regulated genes, including *Period* (*Per*) and *Cryptochrome* (*Cry*). The genomic binding sites of BMAL1 span thousands of locations, many of which are associated with carbohydrate and lipid metabolism [8,9]. Mice with systemic *Bmal1* deficiency lose behavioral rhythms, have abnormalities in lipid and carbohydrate metabolism, and manifest ectopic fat deposition [10].

The kidneys perform various functions, including excretion, maintenance of fluid and electrolyte balance, blood pressure control via the renin–angiotensin–aldosterone system, and production of hormones, such as erythropoietin and renin [11,12,13]. The kidneys express the second-highest number of rhythmic genes among all tissues [7]. Also, several renal functions, including the glomerular filtration, renal blood flow, urine production, and electrolyte excretion, fluctuate in a 24 h cycle [14,15]. According to epidemiological studies, circadian rhythm abnormalities, such as sleep disorders and excessive daytime sleepiness, are particularly frequent in patients with chronic kidney disease (CKD) [16,17]. Furthermore, reduced amplitude and phase disruption in clock gene expression rhythms have been reported in a mouse CKD model [18]. These results suggest that disruption of the circadian clock system may contribute to disease pathogenesis. However, the role of clock genes in the recovery from acute kidney injury (AKI) caused by ischemia–reperfusion injury (IRI) remains unclear.

In this study, we demonstrated that tamoxifen (TAM)-inducible global *Bmal1* knockout (BKO) mice exhibited increased renal lipid accumulation and fibrosis after IRI. This deterioration in renal function and accumulation of lipids resulted from decreased expression of *Pparα* in the kidneys of BKO mice. These findings suggest that BMAL1 plays a protective role in post-AKI renal repair by regulating lipid metabolism.

## 2. Results

### 2.1. Renal Ischemia–Reperfusion Injury (IRI) Disrupts Renal Functions and Alters the Circadian Pattern of Clock Gene Expression

**Figure 1:**
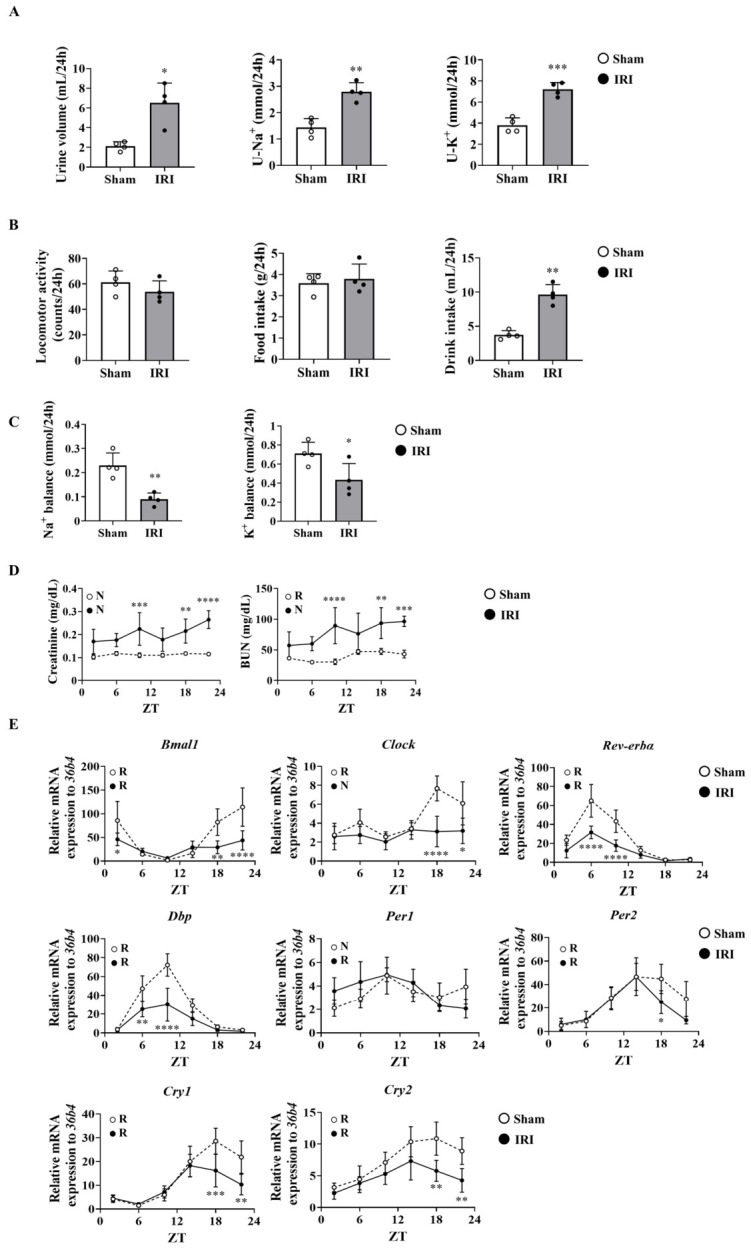
Renal ischemia–reperfusion injury (IRI) disrupted renal function and altered the circadian pattern of clock gene expression in C57BL/6J mice on day 14 post-IRI. (**A**) The urine was collected continuously over a 24 h period. Twenty-four-hour cumulative urine volume, urinary excretion of Na^+^, and urinary K^+^ excretion (*n* = 4 per group). (**B**) Twenty-four-hour locomotor activity, food intake, and water intake (*n* = 4 per group). (**C**) Twenty-four-hour Na^+^ and K^+^ balance (*n* = 4 per group). In (**C**,**D**), samples were collected at each time point. (**D**) Serum creatinine and blood urea nitrogen (BUN) levels in the mice (*n* = 4–5 per group at each time point). (**E**) Clock gene expression in the kidneys of mice (*n* = 4–5 per group at each time point). Data are presented as the mean ±  SD. Data in (**A**–**C**) were compared using an unpaired Student’s *t*-test with Welch’s correction. * *p*  <  0.05, ** *p*  <  0.01, and *** *p* < 0.001, relative to Sham. Data in (**D**,**E**) were compared using two-way ANOVA with Bonferroni’s post hoc test. * *p*  <  0.05, ** *p*  <  0.01, *** *p*  <  0.001, and **** *p* < 0.0001, relative to Sham at the same time point. Detailed statistical results, including F-values, degrees of freedom, and *p*-values for all comparisons, are provided in Table S1. Rhythmicity was calculated using CircWave software version 1.4. R, rhythmic expression (*p*  <  0.05); N, non-rhythmic expression (*p*  >  0.05).

In a first set of experiments, the effects of IRI on the renal functions in C57BL/6J mice were analyzed. The samples were collected on day 14 post-IRI. Day 14 was selected because it corresponds to the repair/remodeling phase in this model [19]. IRI increased the daily urine output and urinary Na^+^ and K^+^ excretion (Figure 1A). Locomotor activity and food intake were comparable between groups, whereas water intake was increased in IRI-treated mice (Figure 1B). Although dietary Na^+^ and K^+^ intake did not differ between groups, the Na^+^ and K^+^ balances were significantly lower in the IRI group, consistent with the increased urinary excretion of these electrolytes (Figure 1C). Then, to analyze the circadian rhythms of renal injury markers and clock gene expression following IRI, mice were dissected at 4 h intervals. Serum creatinine and blood urea nitrogen (BUN) levels were also remarkably elevated in the IRI group (Figure 1D). The gene expression levels of clock genes, except for *Per2* and *Cry2*, were significantly altered in the kidneys of IRI-treated mice (Figure 1E).

### 2.2. Global Bmal1 Deletion Exacerbated IRI-Induced Kidney Damage

The results shown in Figure 1 demonstrate that renal IRI alters the expression pattern of clock genes, including *Bmal1* (Figure 1). Thus, to elucidate the role of BMAL1 in the progression of IRI-induced dysfunction and subsequent recovery, we generated and analyzed the TAM-inducible global *Bmal1* knockout (BKO) mice. This mouse model avoids the developmental effects of *Bmal1* deficiency [20]. TAM-dependent *Bmal1* deletion in the tissues of mice was confirmed by PCR tissue genotyping and Western blotting (Figure S1A,B). There was no difference in body weight, kidney weight, liver weight, and white adipose tissue (WAT) weight between the two types of mice (Figure S1C). Clock gene expression analysis demonstrated that the expressions of *Bmal1* and its target genes, such as *Dbp* and *Rev-erbα*, decreased at **Zeitgeber Time** (ZT) 10 (Figure S1D). Also, diurnal changes in urine volume and K^+^ excretion were diminished in BKO mice (Figure S1E). Diurnal variation in Na^+^ excretion was reduced, although the decrease was not statistically significant (Figure S1E). There was no difference in daily locomotor activity between the two genotypes (Figure S1F). Regarding food and water intake, BKO mice consumed more than *Bmal1*^flox/flox^ mice during ZT0–12, but no difference was observed between the two genotypes during ZT12–24 (Figure S1F).

**Figure 2:**
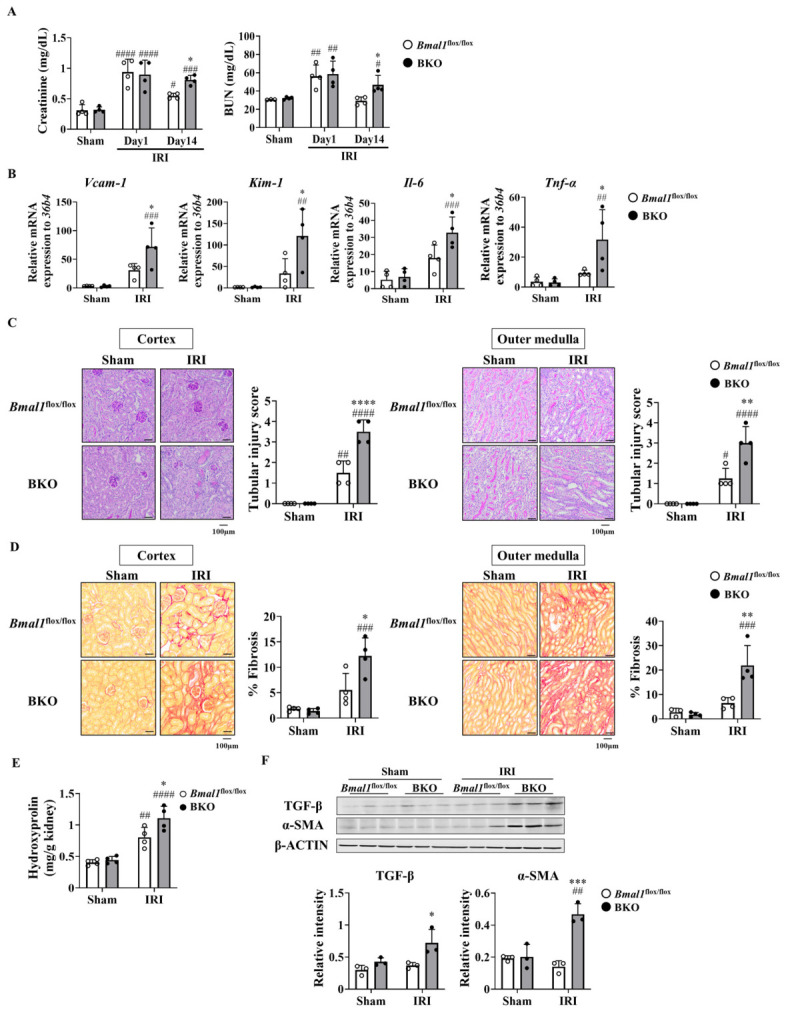
Global *Bmal1* deletion exacerbated IRI-induced kidney injury. *Bmal1*^flox/flox^ and BKO mice were subjected to either a sham operation or IRI (*n* = 4 per group). (**A**) Serum creatinine and BUN levels in mice on days 1 and 14 post-IRI. (**B**) Expression of factors associated with renal impairment in the kidneys of mice on day 14 post-IRI. (**C**) Representative periodic acid–Schiff (PAS)-stained images and corresponding quantification of tubular injury scores in the renal cortex (**left**) and outer medulla (**right**) on day 14 post-IRI. (**D**) Representative Picrosirius red-stained images and corresponding quantification of fibrotic areas in the renal cortex (**left**) and outer medulla (**right**) on day 14 post-IRI. (**E**) Hydroxyproline levels in the kidneys of mice on day 14 post-IRI. (**F**) Top: representative Western blots of α-smooth muscle actin (α-SMA) and transforming growth factor-β (TGF-β). β-ACTIN was used as a loading control. Bottom: the relative intensities of the bands were analyzed using ImageJ software version 1.54d. Data are represented as the mean  ±  SD and compared using the two-way ANOVA with Tukey’s post hoc test. * *p*  <  0.05, ** *p*  <  0.01, *** *p * <  0.001, and **** *p* < 0.0001, relative to *Bmal1*^flox/flox^ mice with the same surgery. ^#^ *p*  <  0.05, or ^##^ *p*  <  0.01, ^###^ *p*  <  0.001, and ^####^ *p * <  0.0001, relative to mice of the same genotype that underwent sham surgery. Detailed statistical results, including F-values, degrees of freedom, and *p*-values for all comparisons, are provided in Table S1.

To understand the role of BMAL1 in kidney damage progression and repair, *Bmal1*^flox/flox^ and BKO mice were subjected to IRI, and pathological changes were analyzed on days 1 and 14 post-IRI. On day 1, the creatinine and BUN levels were elevated in both genotypes. However, by day 14, these marker levels had notably decreased in *Bmal1*^flox/flox^ mice, whereas they maintained high levels, showing a little decrease, in BKO mice (Figure 2A). IRI strongly induced the expression of *Vcam-1*, the chronic tubular injury marker, *Kim-1*, the renal injury marker, and inflammation-related factors such as *Il-6* and *Tnf-α* in the kidneys of both genotypes on day 14 (Figure 2B). The extent of expression induction was more pronounced in BKO mice (Figure 2B). The tubular damage scores in the renal cortex and the outer medulla obtained by PAS staining were markedly higher in BKO mice than in *Bmal1*^flox/flox^ mice (Figure 2C). The fibrotic areas in the renal cortex and the outer medulla of BKO mice were greater, as determined by Picrosirius red staining (Figure 2D). Consistently, renal hydroxyproline content, a biochemical index of collagen deposition, was markedly increased in BKO mice compared to *Bmal1*^flox/flox^ mice post-IRI (Figure 2E). Furthermore, the renal levels of TGF-β and α-SMA were markedly higher in BKO mice than in *Bmal1*^flox/flox^ mice (Figure 2F). These results indicate that global BMAL1 deficiency promotes the development of renal repair defects, fibrosis, and inflammation after IRI.

### 2.3. Global Bmal1 Deletion Induces Ectopic Fat Accumulation in the Kidneys After IRI

**Figure 3:**
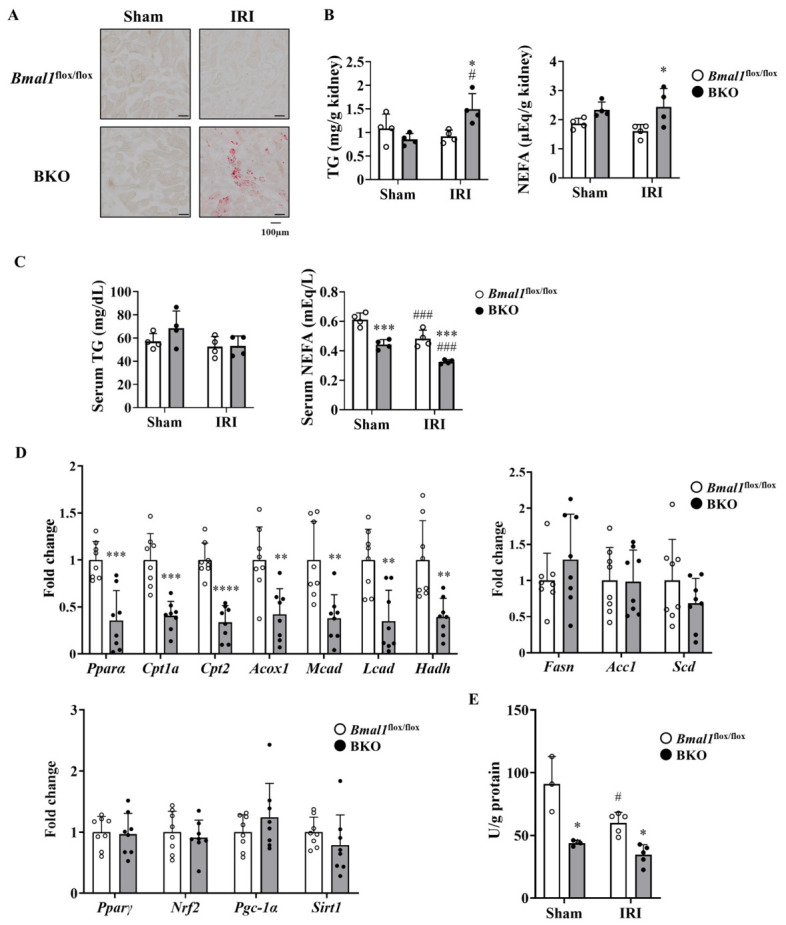
Global *Bmal1* deletion induced ectopic lipid accumulation in the kidneys after IRI. *Bmal1*^flox/flox^ and BKO mice were analyzed on day 14 post-IRI. (**A**) Representative image of Oil Red O staining of the kidneys of mice on day 14 post-IRI. (**B**) Renal triglycerides (TG) and non-esterified fatty acids (NEFA) levels in mice (*n* = 4 per group). (**C**) Serum TG and NEFA levels in mice (*n* = 4 per group). (**D**) Gene expression of factors involved in β-oxidation (**left**), lipogenesis (**right**), and related metabolic regulators (**bottom**) in the kidneys of mice on day 14 post-IRI (*n* = 8 per group). (**E**) The enzymatic activity of β-hydroxyacyl-CoA dehydrogenase (β-HAD) in the kidneys of mice on day 14 post-IRI (*n* = 3–4 per group). Data are represented as the mean ±  SD. Data in (**B**,**C**,**E**) were compared using the two-way ANOVA with Tukey’s post hoc test. * *p*  <  0.05 and *** *p * <  0.001 relative to *Bmal1*^flox/flox^ mice with the same surgery. ^#^ *p * <  0.05 and ^###^ *p * <  0.001 relative to mice of the same genotype that underwent sham surgery. Data in (**D**) were compared using an unpaired Student’s *t*-test with Welch’s correction. ** *p* <  0.01, *** *p * <  0.001 and **** *p  *<  0.0001 relative to *Bmal1*^flox/flox^ mice. Detailed statistical results, including F-values, degrees of freedom, and *p*-values for all comparisons, are provided in Table S1.

In addition to fibrosis and inflammation, several studies have reported renal lipid accumulation in patients with CKD, and this lipid metabolism abnormality is involved in its pathogenesis [21,22,23]. Also, BMAL1 regulates lipid metabolism in various tissues, including the kidney [24,25,26]. Therefore, to investigate the factors that exacerbate renal impairment following IRI in BKO mice, we analyzed renal lipid metabolism. As shown in Figure 3A, IRI induced triglyceride (TG) deposition in the kidneys of BKO mice (Figure 3A,B). The renal NEFA level was higher in BKO mice than in *Bmal1*^flox/flox^ mice (Figure 3B). On the other hand, serum fatty acid levels measured at ZT10 showed no significant change in triglyceride levels, although free fatty acid (FFA) levels decreased significantly (Figure 3C). The expression levels of genes related to lipid oxidation factors examined were all reduced in the kidneys of BKO mice compared to those in *Bmal1*^flox/flox^ mice (Figure 3D). In contrast, no differences were observed in the genes related to lipid synthesis between the two genotypes (Figure 3D). The expression levels of other metabolic regulators related to lipid metabolism, including *Pparγ*, *Nrf2*, *Pgc-1α*, and *Sirt1*, were also comparable between the kidneys of BKO and *Bmal1*^flox/flox^ mice (Figure 3D). Consistent with these gene expression changes, the enzymatic activity of β-hydroxyacyl-CoA dehydrogenase (β-HAD), a crucial enzyme in mitochondrial fatty acid β-oxidation, was markedly decreased in the BKO mouse kidneys (Figure 3E).

### 2.4. Administering Fenofibrate Improved Lipid Metabolism Abnormalities and Renal Dysfunction in BKO Mice

**Figure 4:**
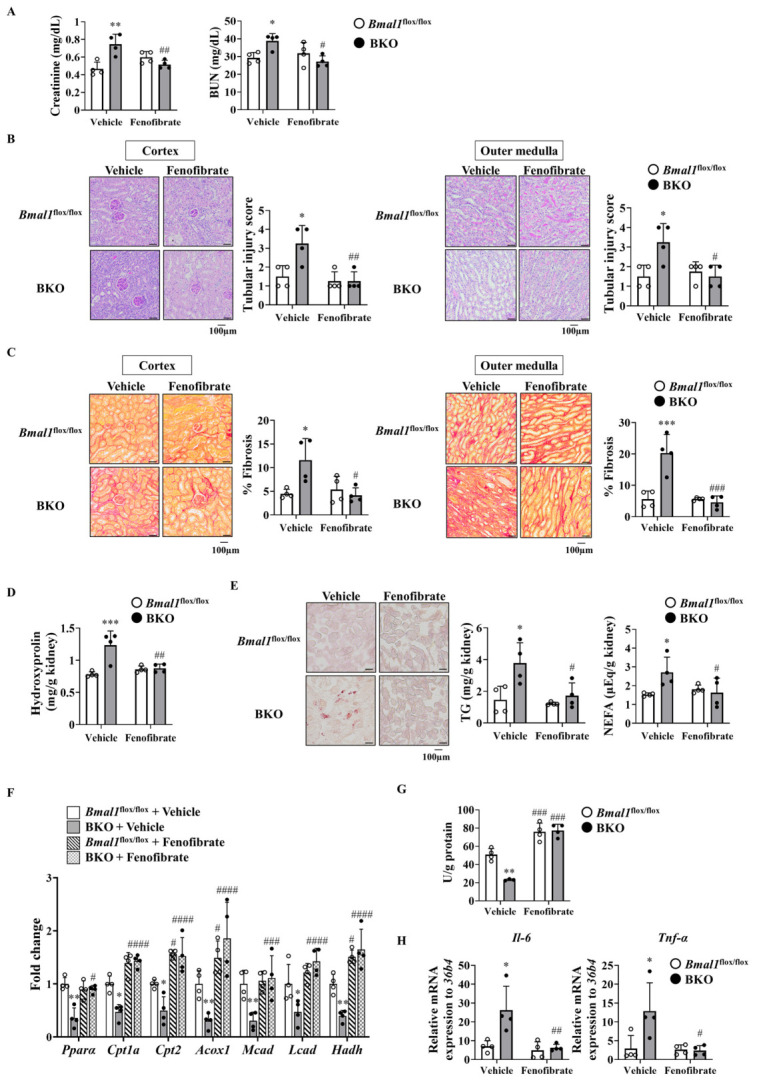
Administering fenofibrate improved lipid metabolism abnormalities and renal dysfunction in BKO mice. *Bmal1*^flox/flox^ and BKO mice were administered vehicle or fenofibrate (50 mg/kg) and analyzed on day 14 post-IRI (*n* = 4 per group). (**A**) Serum creatinine and BUN levels in mice. (**B**) Representative PAS-stained images and corresponding quantification of tubular injury score in the renal cortex (**left**) and outer medulla (**right**) on day 14 post-IRI. (**C**) Representative Picrosirius red-stained images and corresponding quantification of fibrotic areas in the renal cortex (**left**) and outer medulla (**right**) on day 14 post-IRI. (**D**) Hydroxyproline levels in the kidneys of mice. (**E**) Left: representative image of Oil Red O staining of the kidneys of mice on day 14 post-IRI. Right: renal TG and NEFA levels in mice. (**F**) Gene expression of factors involved in β-oxidation in the kidneys of mice. (**G**) The enzymatic activity of β-HAD. (**H**) Expression of factors associated with inflammation. Data are represented as the mean  ±  SD and were compared using two-way ANOVA with Tukey’s post hoc test. * *p*  <  0.05, ** *p*  <  0.01, and *** *p*  <  0.001, relative to *Bmal1*^flox/flox^ mice that received same treatment. ^#^ *p*  <  0.05, ^##^ *p*  <  0.01, ^###^ *p*  <  0.001, and ^####^ *p*  <  0.0001, relative to the vehicle-treated mice of the same genotype. Detailed statistical results, including F-values, degrees of freedom, and *p*-values for all comparisons, are provided in Table S1.

PPARα plays a role in a wide range of functions, including the suppression of inflammation and fibrosis, and the regulation of lipid metabolism [27]. Therefore, to confirm whether decreased *Pparα* expression observed in Figure 3D is involved in the delayed recovery of renal function after IRI in BKO mice, fenofibrate, a PPARα agonist, was administered to BKO mice [28]. Following drug administration, the excessively elevated renal injury marker values and tubular injury scores in the renal cortex and medulla due to *Bmal1* deficiency decreased to levels comparable to those in the *Bmal1*^flox/flox^ group (Figure 4A,B). The increased fibrosis observed in the renal cortex and medulla of BKO mice was also reduced to levels comparable to those in *Bmal1*^flox/flox^ mice after treatment with fenofibrate (Figure 4C). Consistently, the renal hydroxyproline content was elevated in BKO mice post-IRI, and it was reduced by fenofibrate to levels comparable to those in *Bmal1*^flox/flox^ mice (Figure 4D). Although vehicle-administered BKO mouse kidneys had markedly higher levels of TG and NEFA than those of vehicle-administered *Bmal1*^flox/flox^ mice, this difference was eliminated by fenofibrate administration (Figure 4E). In BKO mice, the decreased expression of fatty acid oxidation-related genes was rescued by the administration of fenofibrate (Figure 4F). Along with the restoration of gene expression, fatty acid oxidation activity also recovered (Figure 4G). Furthermore, the expression levels of inflammatory markers, which had been elevated in the BKO mice kidney, decreased to levels comparable to those in the *Bmal1*^flox/flox^ mice kidney following fenofibrate administration (Figure 4H).

### 2.5. Pparα Is a Target Gene of BMAL1 in the Kidneys

**Figure 5:**
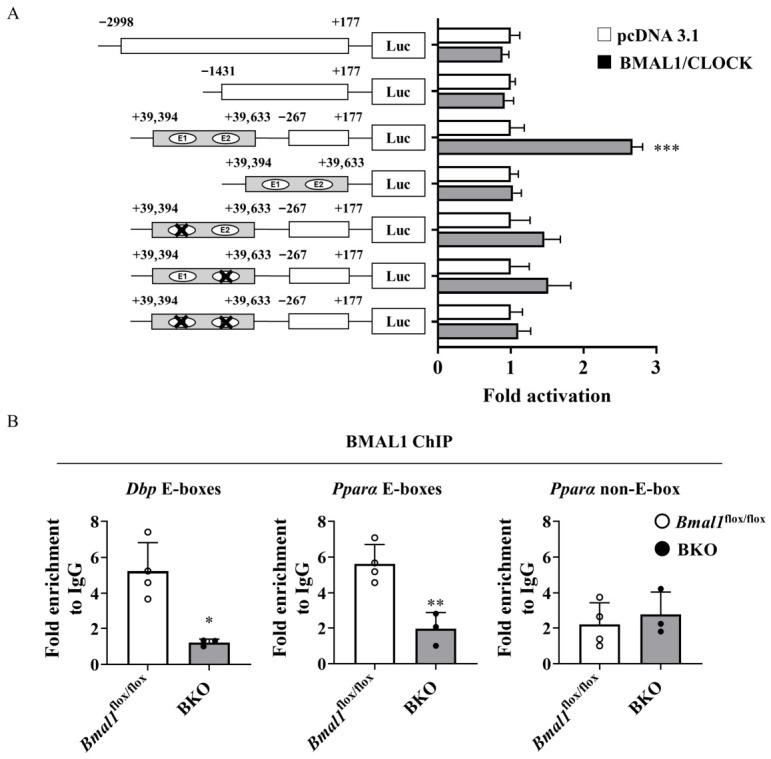
*Pparα* is a target gene of BMAL1 in the kidneys. (**A**) Luciferase activity in HEK293 cells transfected with the reporter plasmids containing the *Pparα* promoter or its mutant variant (× indicates the site of mutation introduced into the E-box) in the presence of BMAL1/CLOCK or empty expression vector (pcDNA3.1) (*n* = 3 per group). Normalized luciferase activity in cells transfected with empty expression vector (pcDNA3.1) was arbitrarily set at 1. Data are presented as the mean ± SD. Data in the panel were compared using an unpaired Student’s *t*-test with Welch’s correction. Significance: *** *p* < 0.001 relative to cells transfected with empty expression vector. (**B**) Chromatin immunoprecipitation (ChIP)–quantitative polymerase chain reaction (qPCR) analysis of the interaction between BMAL1 and the enhancer box (E-box) in the mouse *Pparα* region in the kidney (*n* = 3–4 per group). Non-immune IgG samples were used to normalize the qPCR results for each ChIP sample. The *Dbp* E-box region and *Pparα* non-E-box region were used as positive and negative controls, respectively. Data are presented as the mean ± SD. Data in the panel were compared using an unpaired Student’s *t*-test with Welch’s correction. Significance: * *p*  <  0.05 and ** *p*  <  0.01, relative to *Bmal1*^flox/flox^ mice.

Previous studies have demonstrated that *Pparα* is regulated by BMAL1/CLOCK in the liver [29,30]. To confirm whether BMAL1 can mediate the transactivation of the mouse *Pparα* gene in the kidney, we cloned the 5′ promoter region (−2998 to +177 bp and −1431 to +177 bp) of the mouse *Pparα* gene. As shown in Figure 5A, BMAL1/CLOCK had no effect on the *Pparα* promoter activity as judged by reporter gene assay. These results are consistent with previous findings indicating that no BMAL1 binding site exists within 8 kb of the 5′ flanking region of the mouse *Pparα* gene [28]. *Pparα* inspection revealed that the second intron of the *Pparα* gene contained two perfect E-boxes and four E-box-like motifs within 90 bases [29]. The addition of this region containing E-boxes and E-box-like motifs induced BMAL1/CLOCK-dependent transcriptional activity (Figure 5A). Introduction of a mutation in this E-box resulted in loss of responsiveness to the BMAL1/CLOCK (Figure 5A). The recruitment of BMAL1 to *Pparα*/E-box in the genome was confirmed using a chromatin immunoprecipitation (ChIP) assay. In this assay, the E-box located in the first intron of the mouse DBP gene and part of the mouse Pparα gene, which lacks an E-box, were used as the positive and negative controls, respectively. As shown in Figure 5B, BMAL1 was observed in the intronic E-box-enriched region of the *Pparα* gene in the kidneys of *Bmal1*^flox/flox^ mice, but not in those of BKO mice (Figure 5B).

## 3. Discussion

In a first set of experiments, we confirmed that the circadian pattern of clock genes was altered in the kidneys of mice after IRI (Figure 1E). Previous reports using in vivo or in vitro experimental models have demonstrated a correlation between reduced BMAL1 expression and worsening renal dysfunction [31,32]. However, it remains unclear whether decreased BMAL1 expression causes worsening of dysfunction during the acute phase or delays recovery from injury. Furthermore, it has not yet been elucidated which target genes of the transcription factor BMAL1 regulate renal function. We demonstrated that the loss of *Bmal1* exacerbated renal dysfunction, tubular injury, fibrosis, and inflammation after IRI (Figure 2A–F). In BKO mice, changes in serum creatinine and BUN values during the acute phase (24 h post-IRI) were similar to those in *Bmal1*^flox/flox^ mice (Figure 2A). However, differences in renal dysfunction, fibrosis, and inflammation became more apparent on day 14 post-IRI (Figure 2A–F). These findings indicate that the role of BMAL1 is not to protect the tissue from acute IRI-induced injury but to accelerate the repair phase. It should also be noted that 30 min of ischemia used in this study corresponds to relatively severe injury, and this may have caused a “ceiling effect” that masks further BMAL1-dependent differences during the acute phase. Therefore, we do not rule out the possibility that BMAL1 plays other roles during IRI.

Prior studies on the role of BMAL1 in renal IRI have largely focused on the acute phase within 24 h of reperfusion [31,33,34]. In contrast, the role of BMAL1 during the post-AKI repair phase remains unclear. In addition, whereas previous studies primarily examined endogenous BMAL1 changes or BMAL1 manipulation in non-inducible settings [31,33,34], our study used an adult-onset inducible knockout model. Differences in the timing and extent of BMAL1 loss relative to IRI may therefore also contribute to the distinct temporal pattern observed here. Mechanistically, previous studies have implicated the SIRT1/PGC-1α and NRF2/ARE axis in BMAL1-dependent protection during the acute phase of renal IRI [31,33,34]. In our study, however, *Pgc-1α* and *Nrf2* expression levels were comparable between genotypes on day 14 post-IRI, whereas *Pparα* and its downstream fatty acids oxidation (FAO)-related genes were markedly reduced in BKO mouse kidneys. Recent network analysis has revealed that PPARα is one of the most important transcription factors playing a protective role in human kidney injury [35]. These findings suggest that BMAL1-PPARα signaling may play a more prominent role during post-IRI repair in our model. Consistent with this interpretation, reduced PPARα/FAO activity has been shown to promote lipid accumulation and subsequent tubulointerstitial fibrosis after renal injury [23,25], supporting a model in which impaired PPARα signaling contributes to delayed repair in BKO mice.

Renal triglyceride and NEFA accumulation following IRI in BKO mice closely resembles the pathology observed in human chronic kidney disease (CKD) (Figure 3A,B) [21]. In the injured kidney, such ectopic lipid deposition is likely to be more than a simple metabolic byproduct, because excess intracellular lipid can promote lipotoxic stress, inflammatory signaling, and fibrotic remodeling. This ectopic fat accumulation is likely due in part to reduced FAO activity in the kidneys of BKO mice (Figure 3E). This interpretation is further supported by the reduced expression of *Pparα* and its downstream fatty acid oxidation-related genes in the kidney (Figure 3D). Several reports showed that a reduction in PPARα/FAO axis activity induces fibrosis following lipid accumulation in the kidneys after IRI [36,37]. PPARα regulates not only lipid oxidation but also inflammation during renal injury [38,39]. Reduced PPARα activity may provide a mechanistic link between BMAL1 deficiency and the lipotoxicity, inflammation, and fibrosis observed during delayed recovery after IRI. Indeed, administration of fenofibrate, a PPARα agonist, suppressed lipid accumulation, fibrosis, and inflammation following IRI in BKO mice and improved renal function (Figure 4A–H). The results from *Pparα* knockout mice suggest that the effects of fibrates on fibrosis and inflammation, as well as improvements in lipid metabolism, are dependent on PPARα activity [40,41]. Therefore, while it cannot be completely ruled out that part of the effect of fenofibrate treatment is due to its pharmacological effects, the BMAL1-PPARα axis is an important determinant in the recovery from IRI-induced kidney injury. Although we focused on PPARα as a BMAL1 target in this study, other metabolic regulators involved in lipid metabolism, mitochondrial function, and renal repair may also contribute to the observed phenotype. However, our findings do not support broad transcriptional alteration in these pathways in the kidneys of BKO mice. Rather, impaired PPARα signaling appears to be the predominant alteration in this model. From a molecular regulation perspective, this study demonstrated that, as in the liver, *Bmal1* directly binds to the E-box neighboring regions of the *Pparα* gene and drives its expression in mouse kidneys (Figure 5A,B). Taken together, these findings suggest that BMAL1 contributes to renal repair by regulating *Pparα* expression and that the disruption of this pathway may promote lipotoxicity, inflammation and fibrosis.

Unlike conventional *Bmal1* KO mice with developmental and behavioral issues [42,43,44], the inducible BKO model used here allowed us to examine the impact of adult-onset *Bmal1* deletion while minimizing these confounding effects (Figure S1C,F). No notable differences were observed in the daily activity levels, food intake, or water consumption between BKO mice and control mice (Figure S1F). Ectopic fat accumulation in the kidneys after IRI was observed in BKO mice. The accumulation of ectopic fat in conventional *Bmal1* KO mice results from the release of free fatty acids from adipose tissue [10]. However, BKO mice show no change in WAT tissue weight and serum lipid levels (Figure 3C and Figure S1C). Therefore, it is unlikely that the renal fat accumulation in BKO mice is attributable to lipid influx from other organs.

In conclusion, this study identifies the BMAL1-PPARα axis as a key regulator of renal repair after AKI. From a clinical perspective, these findings suggest that identifying individuals with low clock function and optimizing BMAL1-PPARα axis activity may help prevent AKI chronicity.

## 4. Materials and Methods

### 4.1. Animal Studies

To generate tamoxifen (TAM) (Sigma-Aldrich, St. Louis, MO, USA)-inducible global *Bmal1* knockout mice (BKO), *Bmal1*^flox/flox^ mice were crossed with B6.Cg-Tg(CAG-cre/Esr1*)5Amc/J mice (The Jackson Laboratory, Bar Harbor, ME, USA; stock number 004682), in which Cre-mediated recombination is induced systemically by TAM administration. Male mice (16 to 20 weeks old) were randomly grouped and used in the experiment without any exclusion. Littermates negative for Cre transgenes (*Bmal1*^flox/flox^) were used as experimental controls. To induce Cre activity in mice, TAM (5 mg) was administered orally for 5 consecutive days. All animals were maintained as described previously [10]. All animals were maintained in groups of 3 to 5 animals per cage under a 12 h light-dark cycle at 23 ± 1 °C and 50 ± 10% relative humidity. Food and water were provided ad libitum. C57BL/6J mice were purchased from Sankyo Labo Service (Tokyo, Japan). The experimental methods and design adhered to the Animal Research: Reporting of In Vivo Experiments (ARRIVE) guidelines. The experimental protocol was approved by the Nihon University Animal Care and Use Committee (approval no. AP19PHA007-2 dated 18 June 2019 & approval no. AP24PHA043-1 dated 19 February 2025) and executed according to the relevant guidelines and regulations. Sample sizes were determined based on our previous experience with this IRI model, preliminary observations, and published studies employing comparable endpoints (e.g., histology, qPCR, serum biochemistry). No formal a priori sample size calculation was performed.

### 4.2. Metabolic Studies

The mice were individually caged and acclimated for 2 days before measurements were taken. Locomotor activity, daily food consumption, and water intake were determined using the OsyletPro PhysiocageSystem with Metabolism software version 3.0 (PANLAB S.L.U., Barcelona, Spain). To measure 24 h cumulative urine volume, urinary excretion of Na^+^, and urinary K^+^ excretion, urine samples were collected continuously during the measurement period, and urinary Na^+^ and K^+^ excretion were determined. Na^+^ and K^+^ intakes were calculated from daily food consumption and the electrolyte content of the diet. Twenty-four-hour Na^+^ and K^+^ balances were then calculated by subtracting urinary Na^+^ and K^+^ excretion from the corresponding intake values.

### 4.3. Renal Ischemia–Reperfusion Injury (IRI) Model

A lateral abdominal incision was made to expose the kidneys of the mice, and both renal arteries were clamped using stainless-steel arterial clamps (Natsume Manufacturing, Tokyo, Japan) for 30 min [45]. The clips were removed immediately after the elapse of the ischemia time, and reperfusion was initiated. Sham-operated mice underwent the same procedures without renal artery clamping. On day 14 after surgery, sham-operated and IRI-treated C57BL/6J mice were euthanized at 4 h intervals (ZT2, 6, 10, 14, 18, and 22), and samples were collected at each time point.

### 4.4. Biochemical Analysis

The triglyceride (TG) (EKF Diagnostics-Stanbio Laboratory, Boerne, TX, USA), non-esterified fatty acid (NEFA), creatinine (Fujifilm Wako Pure Chemical Co., Ltd., Osaka, Japan), and blood urea nitrogen (BUN) (BioAssay Systems, Hayward, CA, USA) levels were determined using commercial assay kits. Urinary sodium, urinary potassium, serum sodium, and serum potassium concentrations were measured using the ion-selective electrode method at Oriental Yeast Co., Ltd. (Tokyo, Japan). Hydroxyproline levels were quantified using the chloramine-T/Ehrlich’s reagent method with trans-4-hydroxy-L-proline (TCI, Tokyo, Japan).

### 4.5. Histological Analysis

The kidneys were fixed, embedded in paraffin wax, and sliced into 5 µm sections. To calculate the renal damage score, ten views of the renal cortex and outer medulla (×200) were randomly selected separately per sample using periodic acid–Schiff (PAS)-stained specimens, and the extent of tubular damage was evaluated using a 5-point scale based on the percentage of damaged tubules showing tubular dilatation, intratubular cast formation, loss of the brush border, and epithelial cell injury: 0 (0–5%); 1 (5–25%); 2 (25–50%); 3 (50–75%); and 4 (75–100%) [46]. Interstitial fibrosis was assessed using Picrosirius red staining, after which the fibrotic area was measured separately in the cortex and medulla by Image J software (National Institutes of Health, Bethesda, MD, USA). To evaluate neutral lipid accumulation, frozen sections were stained with Oil Red O (Sigma-Aldrich, St. Louis, MO, USA for 30 min, rinsed with 60% isopropanol, and counterstained with hematoxylin [29].

### 4.6. Preparation of Tissue Extract

Tissue samples were added to a RIPA buffer (50 mM Tris-HCl [pH 8.0], 150 mM NaCl, 0.1% SDS, 1% NP-40, and 0.5% sodium deoxycholate) containing a protease inhibitor cocktail (Fujifilm Wako Pure Chemical Co., Ltd., Osaka, Japan) and were homogenized using a Dounce grinder. Protein concentrations were determined using a bicinchoninic acid (BCA) protein assay (Takara Bio Inc., Shiga, Japan).

### 4.7. Western Blotting

Proteins were separated by size using sodium dodecyl sulfate-polyacrylamide gel electrophoresis (SDS-PAGE), transferred onto membranes, and probed with the relevant antibodies. Immunoreactive proteins were visualized using enhanced chemiluminescence detection reagents (Thermo Fisher Scientific, Waltham, MA, USA, and Cytiva, Marlborough, MA, USA). Band intensity was quantified using Image J software version 1.54d (National Institutes of Health, Bethesda, MD, USA). The anti-BMAL1 antibody was purchased from Abcam (Cambridge, UK). Anti-transforming growth factor (TGF)-β and anti-α-smooth muscle actin (SMA) antibodies were purchased from Cell Signaling Technology (Danvers, MA, USA). The anti-β-ACTIN antibody was purchased from ProteinTech Group Inc. (Rosemont, IL, USA).

### 4.8. RNA Isolation and Reverse Transcription Quantitative Polymerase Chain Reaction (qPCR)

Total RNA was extracted from the kidneys using the RNA isoPlus kit (Takara Bio, Shiga, Japan). cDNA was synthesized from the total RNA (1.0 µg) using a reverse transcriptase (Toyobo, Osaka, Japan), and cDNA was amplified using an AriaMx Realtime PCR System (Agilent Technologies, Santa Clara, CA, USA) using GoTaq qPCR Master Mix (Promega, Madison, WI, USA). The mRNA expression levels were normalized to the *36b4* expression levels and are presented as relative levels. The primer sequences are presented in Table 1.

### 4.9. Fenofibrate Administration

Fenofibrate (50 mg/kg; Sigma-Aldrich, St. Louis, MO, USA) suspended in 0.5% (*w*/*v*) carboxymethylcellulose sodium (CMC) solution was administered orally once daily to *Bmal1*^flox/flox^ and BKO mice. Treatment was performed for 15 consecutive days, starting on the day of reperfusion. Mice in the vehicle group received the same volume of 0.5% CMC solution.

### 4.10. Plasmid Construction, Cell Culture and Cell Transfection

The 5′ regulatory region (−2998 to +177 bp, +39,394 to +39,633 bp) of the mouse *Pparα* gene was amplified by PCR using mouse genomic DNA as a PCR template and the following oligonucleotides: PPARα −2998: 5′-CGCGGTACCAGAGGCATAGCCCACTGGGGATG-3′, −1431: 5′-CGCGGTACCTCAATACAGTCTGTCAAACAAAATT-3, −267: 5′-CGCCTCGAGGCATGTGCATTCCGTGACTGAAG-3, +177: 5′-CGCAAGCTTCGTTGAGCTGGTCTAGATCGCACAG-3′, +39,394: 5′-CGCGGTACCGTAAGCACAGGTTTCTTGCG-3′ and +39,633: 5′-CGCGCTAGCACTCTATCTCAAAGAGTAAAGTCG-3′. The PCR-amplified fragment was cloned into the pGL3-basic vector (Promega, Madison, WI, USA). Site-directed mutation was introduced in the 5′ regulatory region (+39,397 to +39,633 bp) of the mouse *Pparα* gene by the PCR overextension method and confirmed by DNA sequencing. HEK293 cells, obtained from Human Science Research Resources Bank (Osaka, Japan), were maintained in Dulbecco’s modified Eagle medium (DMEM) supplemented with 10% fetal bovine serum. HEK293 cells were transfected with the plasmids using FuGene HD (Promega). The cells’ lysates were prepared 16 h later and assayed with a dual luciferase reporter assay system (Promega). The pGL4.75-CMV vector (Promega) was used as a normalization control to correct for variable transfection efficiencies.

### 4.11. Chromatin Immunoprecipitation (ChIP)

The ChIP assay was performed as previously described [47]. Briefly, kidneys from mice were harvested, cross-linked with 1% formaldehyde, and lysed. The tissue extracts were then subjected to immunoprecipitation using an anti-BMAL1 antibody (Abcam, Cambridge, UK). Parallel samples were incubated with non-immune IgG (Fujifilm Wako Pure Chemical Co., Ltd., Osaka, Japan) as a negative control. The DNA region was amplified and quantified using qPCR. Data are expressed as fold enrichment of IgG. The following PCR primers were used: DBP Enhancer box (E-box) +543/+800 (forward: 5′-TGACAGCTCAGTAATTCTCCC-3′, reverse: 5′-CTTGAGGACAGAGTTTAGGTG-3′),

PPARα E-boxes +39,394/+39,633 (forward: 5′-GTAAGCACAGGTTTCTTGCGT-3′, reverse: 5′-ACTCTATCTCAAAGAGTAAAG-3′), PPARα non-E-box −3793/−3733 (forward: 5′-TGGCACCTTGGCCACCTGTT-3′, reverse: 5′-TGTCTGATTGGCTGCTGCGG-3′).

### 4.12. Measurement of 3-Hydroxyacyl CoA Dehydrogenase Activity

Kidney tissues were homogenized in 0.1 M phosphate buffer and centrifuged (15,000× *g*, 15 min). The supernatants were mixed with an assay buffer containing 100 mM triethanolamine, 5 mM EDTA, and 0.45 mM NADH (Calbiochem, San Diego, CA, USA). The reaction was initiated by adding acetoacetyl-CoA (MP Biomedicals, Irvine, CA, USA) at 30 °C, and the enzymatic activity of β-HAD was calculated from the rate of decrease in absorbance at 340 nm [48].

### 4.13. Statistical Analysis

All statistical analyses were performed using GraphPad Prism 10 software (GraphPad Software, San Diego, CA, USA). Continuous data are expressed as the mean ± standard deviation (SD). Differences between the two groups were determined using an unpaired Student’s *t*-test with Welch’s correction. For experiments involving two independent variables, data were analyzed using two-way analysis of variance (ANOVA) followed by Bonferroni’s or Tukey’s post hoc test. Detailed ANOVA stats are summarized in Table S1. Circadian rhythmicity was assessed using the CircWave software version 1.4 (Roelof Hut, University of Groningen, Groningen, The Netherlands) [49].

## Figures and Tables

**Table 1:** Primer sequence.

Primer	Forward 5′–3′	Reverse 5′–3′
*36b4*	AAGCGCGTCCTGGCATTGTC	CCGCAGGGGCAGCAGTGGT
*Acc1*	AATGGTCTCTTTCCGGACCT	GCAAGCCTGTCATCCTCAAT
*Acox1*	CAGGAAGAGCAAGCAAGTGG	CCTTTCTGGCTGATCCCATA
*Bmal1*	ATGCAGAAACACCAAGGAAGG	CCATCCTTAGCACGGTGAGT
*Clock*	TGCCAGCTCATGAAAAGATG	TTGCTGCCTCATCGTTACTG
*Cpt1a*	TTGATCAAGAAGTGCCGGACGAGT	GTCCATCATGGCCAGCACAAAGTT
*Cpt2*	TCCTCGATCAAGATGGGAAC	GATCCTTCATCGGGAAGTCA
*Cry1*	TCCCCTCCCCTTTCTCTTTA	TGAGTCATGATGGCGTCAT
*Cry2*	CTCAGCAGAGAGCTGCCTTT	GACGCAGAATTAGCCTTTGC
*Dbp*	GCATTCCAGGCCATGAGAC	GTTCTTGTACCTCCGGCTCCA
*Fasn*	TGCCTCCCAGCTGCAGGC	GCCCGGTAGCTCTGGGTGTA
*Hadh*	CCACCAGACAAGACCGATTT	TCAATGAGGTATGGCACC
*IL-6*	ACAACCACGGCCTTCCCTACTT	CACGCTTTCCCAGAGAACATGTG
*Kim-1*	TGTCGAGTGGAGATTCCTCGATGG	GGTCTTCCTGTAGCTGTGGGCC
*Lcad*	ATGGCAAAATACTGGGCATC	TCTTGCGATCAGCTCTTTCA
*Mcad*	CGCTCTTAGGACTACTTGCTAACC	ATGGTATTTACATGCAATGGACAG
*Nrf2*	TCACACGAGATGAGCTTAGGGCAA	TACAGTTCTGGGCGGCGAACTTTAT
*Per1*	TCCCTGCTATGTCTCCCATC	GGGGTCACTAAGGGAGAAGG
*Per2*	AGAGTGTGGTGTCCCATC	ATGTGCACTAAGGGAGAAGG
*Pgc-1α*	GCATTCCTCAATTTGAGGAA	AATCAGACCTGACACAACGC
*Pparα*	TCAAGGTGTGGCCCAAGGTTA	CGAATGTTCTCAGAAGCCAGCTC
*Pparγ*	ATGGAGCCTAAGTTTGAGTTTGC	GATGTCCTCGATGGGCTTCAC
*Rev-erbα*	CTTCCGTGACCTTTCTCAGC	CAGCTCCTCCTCGGTAAGTG
*Scd*	TGGGTTGGCTGCTTGTG	GCGTGGGCAGGATGAAG
*Sirt1*	CCTGAGATTGGCACCGATTT	CAGGGAATCCCAGGCAAGTA
*Tnf* *-* *α*	CGTCAGCCGATTTGCTATCT	CGGACTCCGCAAAGTCTAAG
*Vcam-1*	TTACTCCCGGTCATTGAGGAT	ACAGGTCATTGTCACAGCAC

## Data Availability

The datasets used and analyzed during the current study are available from the corresponding author on reasonable request.

## Supplementary Materials

The following supporting information can be downloaded at https://www.mdpi.com/article/10.3390/ijms27094091/s1.

## Abbreviations

The following abbreviations are used in this manuscript:

ACC1	Acetyl-CoA carboxylase 1
ACOX1	Acyl-CoA oxidase 1
AKI	Acute kidney injury
ANOVA	Analysis of variance
ARRIVE	Animal Research: Reporting of In Vivo Experiments
ARNTL	Aryl hydrocarbon receptor nuclear translocator-like protein 1
BKO	Bmal1 knockout
BMAL1	Brain and muscle Arnt-like protein 1
BUN	Blood urea nitrogen
ChIP	Chromatin immunoprecipitation
CKD	Chronic kidney disease
CLOCK	Circadian locomotor output cycles kaput
CMV	Cytomegalovirus
CPT	Carnitine palmitoyltransferase
CRY	Cryptochrome
DBP	D site-binding protein
E-box	Enhancer box
ECL	Enhanced chemiluminescence
eWAT	Epididymal white adipose tissue
FAO	Fatty acid β-oxidation
FAS	Fatty acid synthase
GFR	Glomerular filtration rate
HAD	β-Hydroxyacyl-CoA dehydrogenase
H/R	Hypoxia/reoxygenation
IL-6	Interleukin-6
IRI	Ischemia–reperfusion injury
KIM-1	Kidney injury molecule-1
KO	Knockout
LCAD	Long-chain acyl-CoA dehydrogenase
MCAD	Medium-chain acyl-CoA dehydrogenase
NEFA	Non-esterified fatty acids
NP-40	Nonidet P-40
NRF2	Nuclear factor erythroid 2-related factor 2
PBS	Phosphate-buffered saline
PGC-1α	Peroxisome proliferator-activated receptor gamma coactivator 1alpha
PPARα	Peroxisome proliferator-activated receptor alpha
PPARγ	Peroxisome proliferator-activated receptor gamma
qPCR	Quantitative polymerase chain reaction
REV-ERBα	Reverse erythroblastosis virus alpha
RQ	Respiratory quotient
RORE	ROR response element
SCD1	Stearoyl-CoA desaturase 1
SD	Standard deviation
SDS-PAGE	Sodium dodecyl sulfate-polyacrylamide gel electrophoresis
SIRT1	Sirtuin1
TGF-β	Transforming growth factor-β
TG	Triglyceride
TNFα	Tumor necrosis factor-α
VCAM-1	Vascular cell adhesion molecule-1
WT	Wild type
ZT	Zeitgeber time
