# Supplementary material for: Deletion of Bmal1, a Component of the Molecular Clock, Exacerbates Kidney Damage After Ischemia–Reperfusion by Decreasing Pparα Expression
Source: Int J Mol Sci. 2026 May 2;27(9):4091. doi: 10.3390/ijms27094091 (PMC13163576; doi:10.3390/ijms27094091)
Supplement: Supplementary file 1 [file ijms-27-04091-s001.zip › Figure S1.pdf]

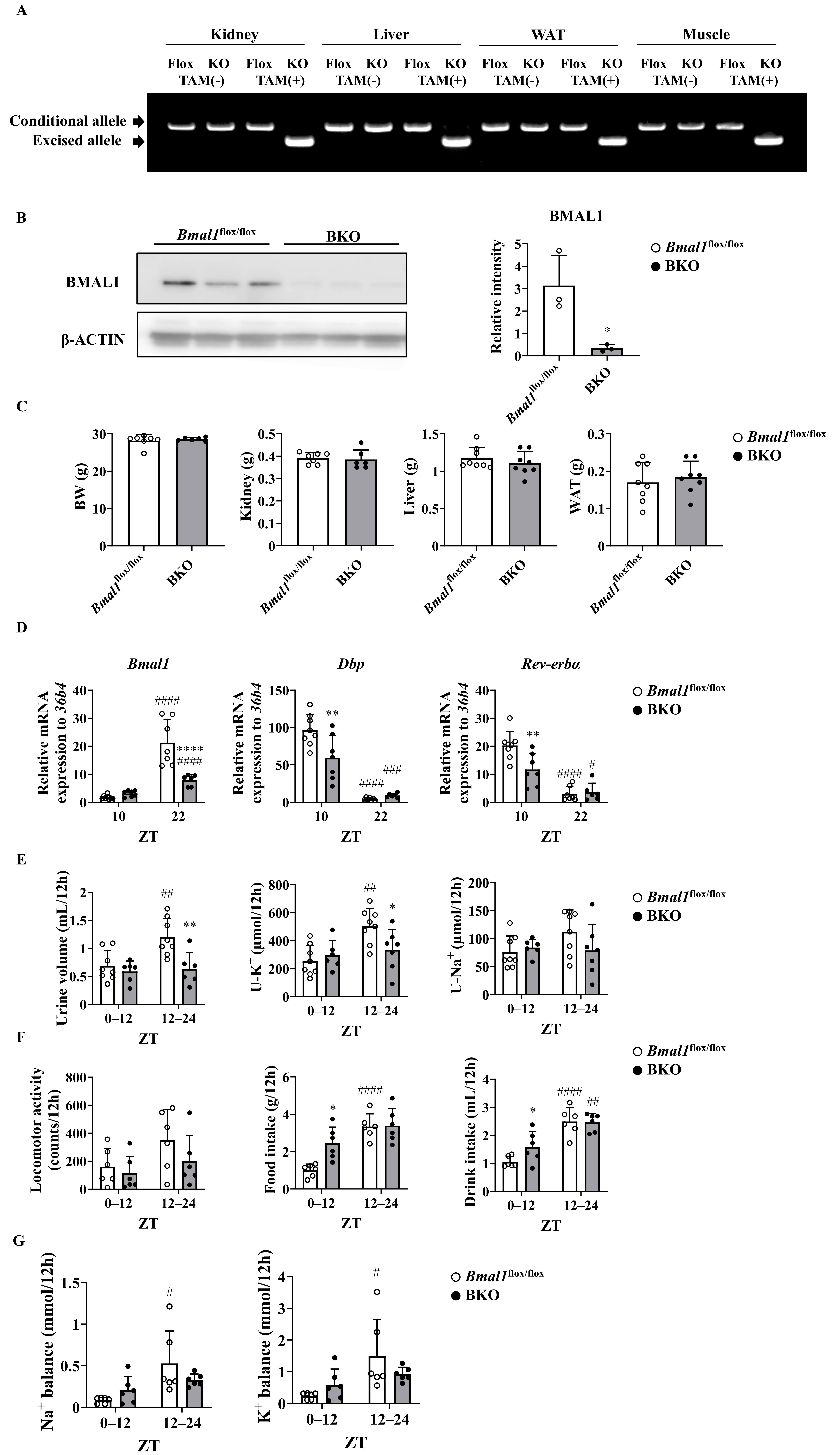

Figure S1. Deletion of *Bmal1* gene has no effects on developmental and behavioral rhythms. (A) PCR product amplified from genomic DNA isolated from the tissues of *Bmal1*$^{flox/flox}$ (Flox) and BKO mice (KO) treated with vehicle (TAM (−)) or tamoxifen (TAM (+)). (B) Representative Western blot of the BMAL1 protein in the kidney. β-ACTIN was used as the loading control. Right, the relative intensities of the bands were analyzed using ImageJ software. (C) Body weight (BW), kidney, liver, and white adipose tissue (WAT) weights (from left to right) (n = 6–7 per group). (D) Gene expression of clock genes in the kidneys of *Bmal1*$^{flox/flox}$ and BKO mice at ZT10 and ZT22 (n = 6–7 per group). (E) Twelve-hour cumulative renal excretion of urine volume, urinary K^+ excretion, and urinary Na^+ excretion of *Bmal1*$^{flox/flox}$ and BKO mice (n = 7–8 per group). (F) Twelve-hour cumulative locomotor activity, food intake, and water intake of *Bmal1*$^{flox/flox}$ and BKO mice (n = 6 per group). (G) Twelve-hour Na^+ and K^+ balance of *Bmal1*$^{flox/flox}$ and BKO mice (n = 6 per group). Data are represented as the mean ± SD. Data in (B and C) were compared using an unpaired Student's t-test with Welch's correction. *$P < 0.05$ relative to *Bmal1*$^{flox/flox}$. Data in (D-G) were compared using two-way ANOVA with Tukey's *post hoc* test. *$P < 0.05$, **$P < 0.01$, and ****$P < 0.001$, relative to *Bmal1*$^{flox/flox}$ at the same time point (D) or the same time phase (E–G). $^{\#}P < 0.05$, $^{\#\#}P < 0.01$, $^{\#\#\#}P < 0.001$, and $^{\#\#\#\#}P < 0.0001$, relative to *Bmal1*$^{flox/flox}$ mice at ZT10 (D) or ZT0-12 (E–G) at the genotype.
