# Supplementary material for: Deletion of Bmal1, a Component of the Molecular Clock, Exacerbates Kidney Damage After Ischemia–Reperfusion by Decreasing Pparα Expression
Source: Int J Mol Sci. 2026 May 2;27(9):4091. doi: 10.3390/ijms27094091 (PMC13163576; doi:10.3390/ijms27094091)
Supplement: Supplementary file 1 [file ijms-27-04091-s001.zip › Table S1.pdf]

**Table S1. Two-way ANOVA statistics.**

| Figure | Outcome | Factor A | Factor B | Interaction F (df1, df2) | Interaction p | Factor A F (df1, df2) | Factor A p | Factor B F (df1, df2) | Factor B p |
|---|---|---|---|---|---|---|---|---|---|
| Fig. 1E | Serum creatinine | Time (ZT) | Surgery | F(5, 42) = 1.91 | 0.1125 | F(5, 42) = 2.37 | 0.0557 | F(1, 42) = 80.87 | < 0.0001 |
| Fig. 1E | BUN | Time (ZT) | Surgery | F(5, 42) = 1.74 | 0.1473 | F(5, 42) = 3.48 | 0.0102 | F(1, 42) = 68.39 | < 0.0001 |
| Fig. 1F | *Bmal1* mRNA | Time (ZT) | Surgery | F(5, 44) = 7.06 | < 0.0001 | F(5, 44) = 20.83 | < 0.0001 | F(1, 44) = 18.85 | < 0.0001 |
| Fig. 1F | *Clock* mRNA | Time (ZT) | Surgery | F(5, 44) = 4.52 | 0.0021 | F(5, 44) = 8.07 | < 0.0001 | F(1, 44) = 23.48 | < 0.0001 |
| Fig. 1F | *Rev-erbα* mRNA | Time (ZT) | Surgery | F(5, 44) = 7.93 | < 0.0001 | F(5, 44) = 51.40 | < 0.0001 | F(1, 44) = 36.98 | < 0.0001 |
| Fig. 1F | *Dbp* mRNA | Time (ZT) | Surgery | F(5, 44) = 8.45 | < 0.0001 | F(5, 44) = 52.30 | < 0.0001 | F(1, 44) = 35.99 | < 0.0001 |
| Fig. 1F | *Per1* mRNA | Time (ZT) | Surgery | F(5, 44) = 2.98 | 0.0211 | F(5, 44) = 5.36 | 0.0006 | F(1, 44) = 0.39 | 0.5343 |
| Fig. 1F | *Per2* mRNA | Time (ZT) | Surgery | F(5, 43) = 2.28 | 0.0636 | F(5, 43) = 24.73 | < 0.0001 | F(1, 43) = 5.26 | 0.0267 |
| Fig. 1F | *Cry1* mRNA | Time (ZT) | Surgery | F(5, 44) = 5.01 | 0.0010 | F(5, 44) = 39.47 | < 0.0001 | F(1, 44) = 12.35 | 0.0010 |
| Fig. 1F | *Cry2* mRNA | Time (ZT) | Surgery | F(5, 44) = 2.17 | 0.0749 | F(5, 44) = 14.89 | < 0.0001 | F(1, 44) = 27.39 | < 0.0001 |
| Fig. 2A | Serum creatinine | Time point | Genotype | F(2, 18) = 2.67 | 0.0962 | F(2, 18) = 37.23 | < 0.0001 | F(1, 18) = 1.78 | 0.1991 |
| Fig. 2A | BUN | Time point | Genotype | F(2, 17) = 1.81 | 0.1947 | F(2, 17) = 16.19 | 0.0001 | F(1, 17) = 3.42 | 0.0821 |
| Fig. 2B | *Vcam-1* mRNA | Surgery | Genotype | F(1, 12) = 5.43 | 0.0380 | F(1, 12) = 29.68 | 0.0001 | F(1, 12) = 5.22 | 0.0413 |
| Fig. 2B | *Kim-1* mRNA | Surgery | Genotype | F(1, 12) = 5.87 | 0.0321 | F(1, 12) = 17.74 | 0.0012 | F(1, 12) = 5.90 | 0.0318 |
| Fig. 2B | *Il-6* mRNA | Surgery | Genotype | F(1, 11) = 3.94 | 0.0728 | F(1, 11) = 25.67 | 0.0004 | F(1, 11) = 4.22 | 0.0646 |
| Fig. 2B | *Tnf-α* mRNA | Surgery | Genotype | F(1, 11) = 4.58 | 0.0555 | F(1, 11) = 9.19 | 0.0114 | F(1, 11) = 3.57 | 0.0855 |
| Fig. 2C | Tubular injury score (cortex) | Surgery | Genotype | F(1, 12) = 24.00 | 0.0004 | F(1, 12) = 150.00 | < 0.0001 | F(1, 12) = 24.00 | 0.0004 |
| Fig. 2C | Tubular injury score (outer medulla) | Surgery | Genotype | F(1, 12) = 13.36 | 0.0033 | F(1, 12) = 78.82 | < 0.0001 | F(1, 12) = 13.36 | 0.0033 |
| Fig. 2D | Sirius red-positive area (cortex) | Surgery | Genotype | F(1, 12) = 8.57 | 0.0127 | F(1, 12) = 35.83 | < 0.0001 | F(1, 12) = 6.61 | 0.0245 |
| Fig. 2D | Sirius red-positive area (outer medulla) | Surgery | Genotype | F(1, 12) = 12.40 | 0.0048 | F(1, 12) = 26.55 | 0.0003 | F(1, 12) = 9.69 | 0.0099 |
| Fig. 2E | Hydroxyproline | Surgery | Genotype | F(1, 12) = 4.19 | 0.0632 | F(1, 12) = 67.81 | < 0.0001 | F(1, 12) = 7.16 | 0.0202 |
| Fig. 2F | TGF-β protein | Surgery | Genotype | F(1, 8) = 1.55 | 0.2484 | F(1, 8) = 4.71 | 0.0618 | F(1, 8) = 8.53 | 0.0193 |
| Fig. 2F | α-SMA protein | Surgery | Genotype | F(1, 8) = 25.42 | 0.0010 | F(1, 8) = 11.39 | 0.0097 | F(1, 8) = 28.53 | 0.0007 |

| Figure | Outcome | Factor A | Factor B | Interaction F (df1, df2) | Interaction p | Factor A F (df1, df2) | Factor A p | Factor B F (df1, df2) | Factor B p |
|---|---|---|---|---|---|---|---|---|---|
| Fig. 3B | Kidney TG | Surgery | Genotype | F(1, 12) = 11.32 | 0.0056 | F(1, 12) = 3.96 | 0.0698 | F(1, 12) = 2.07 | 0.1762 |
| Fig. 3B | Kidney FFA | Surgery | Genotype | F(1, 12) = 0.71 | 0.4145 | F(1, 12) = 0.24 | 0.6332 | F(1, 12) = 10.88 | 0.0064 |
| Fig. 3C | Serum TG | Surgery | Genotype | F(1, 12) = 1.16 | 0.3031 | F(1, 12) = 3.83 | 0.0741 | F(1, 12) = 1.38 | 0.2632 |
| Fig. 3C | Serum NEFA | Surgery | Genotype | F(1, 12) = 0.09 | 0.7710 | F(1, 12) = 36.84 | < 0.0001 | F(1, 12) = 63.89 | < 0.0001 |
| Fig. 3E | β-HAD activity | Surgery | Genotype | F(1, 12) = 3.52 | 0.0852 | F(1, 12) = 11.93 | 0.0048 | F(1, 12) = 39.03 | < 0.0001 |
| Fig. 4A | Serum creatinine | Treatment | Genotype | F(1, 12) = 20.21 | 0.0007 | F(1, 12) = 1.49 | 0.2464 | F(1, 12) = 6.09 | 0.0297 |
| Fig. 4A | BUN | Treatment | Genotype | F(1, 12) = 7.27 | 0.0194 | F(1, 12) = 2.74 | 0.1240 | F(1, 12) = 0.64 | 0.4390 |
| Fig. 4B | Tubular injury score (cortex) | Treatment | Genotype | F(1, 12) = 7.00 | 0.0213 | F(1, 12) = 11.57 | 0.0053 | F(1, 12) = 7.00 | 0.0213 |
| Fig. 4B | Tubular injury score (outer medulla) | Treatment | Genotype | F(1, 12) = 8.73 | 0.0120 | F(1, 12) = 4.91 | 0.0468 | F(1, 12) = 4.91 | 0.0468 |
| Fig. 4C | Sirius red-positive area (cortex) | Treatment | Genotype | F(1, 12) = 8.71 | 0.0121 | F(1, 12) = 5.41 | 0.0383 | F(1, 12) = 4.27 | 0.0610 |
| Fig. 4C | Sirius red-positive area (outer medulla) | Treatment | Genotype | F(1, 12) = 21.50 | 0.0006 | F(1, 12) = 21.38 | 0.0006 | F(1, 12) = 16.16 | 0.0017 |
| Fig. 4D | Hydroxyproline | Treatment | Genotype | F(1, 12) = 13.51 | 0.0032 | F(1, 12) = 5.55 | 0.0363 | F(1, 12) = 15.50 | 0.0020 |
| Fig. 4E | Kidney TG | Treatment | Genotype | F(1, 12) = 4.34 | 0.0593 | F(1, 12) = 6.92 | 0.0219 | F(1, 12) = 10.46 | 0.0072 |
| Fig. 4E | Kidney FFA | Treatment | Genotype | F(1, 12) = 6.36 | 0.0268 | F(1, 12) = 1.65 | 0.2237 | F(1, 12) = 2.96 | 0.1110 |
| Fig. 4F | *Ppara* mRNA | Treatment | Genotype | F(1, 12) = 20.13 | 0.0007 | F(1, 12) = 9.12 | 0.0107 | F(1, 12) = 19.80 | 0.0008 |
| Fig. 4F | *Cpt1a* mRNA | Treatment | Genotype | F(1, 12) = 7.93 | 0.0156 | F(1, 12) = 50.80 | < 0.0001 | F(1, 12) = 5.51 | 0.0369 |
| Fig. 4F | *Cpt2* mRNA | Treatment | Genotype | F(1, 12) = 4.17 | 0.0637 | F(1, 12) = 51.49 | < 0.0001 | F(1, 12) = 6.12 | 0.0293 |
| Fig. 4F | *Acox1* mRNA | Treatment | Genotype | F(1, 12) = 6.91 | 0.0220 | F(1, 12) = 25.51 | 0.0003 | F(1, 12) = 0.66 | 0.4337 |
| Fig. 4F | *Mcad* mRNA | Treatment | Genotype | F(1, 12) = 7.47 | 0.0181 | F(1, 12) = 9.80 | 0.0087 | F(1, 12) = 5.38 | 0.0389 |
| Fig. 4F | *Lcad* mRNA | Treatment | Genotype | F(1, 12) = 7.85 | 0.0160 | F(1, 12) = 19.83 | 0.0008 | F(1, 12) = 1.52 | 0.2412 |
| Fig. 4F | *Hadh* mRNA | Treatment | Genotype | F(1, 12) = 11.11 | 0.0060 | F(1, 12) = 53.11 | < 0.0001 | F(1, 12) = 4.67 | 0.0516 |
| Fig. 4G | β-HAD activity | Treatment | Genotype | F(1, 11) = 15.91 | 0.0021 | F(1, 11) = 116.20 | < 0.0001 | F(1, 11) = 12.76 | 0.0044 |
| Fig. 4H | *Il-6* | Treatment | Genotype | F(1, 12) = 6.43 | 0.0261 | F(1, 12) = 9.98 | 0.0082 | F(1, 12) = 8.71 | 0.0121 |
| Fig. 4H | *Tnf-α* | Treatment | Genotype | F(1, 12) = 5.85 | 0.0324 | F(1, 12) = 6.48 | 0.0257 | F(1, 12) = 5.29 | 0.0403 |

| Figure | Outcome | Factor A | Factor B | Interaction F (df1, df2) | Interaction p | Factor A F (df1, df2) | Factor A p | Factor B F (df1, df2) | Factor B p |
|---|---|---|---|---|---|---|---|---|---|
| Fig. S1 D | *Bmal1* mRNA | Time | Genotype | F(1, 24) = 7.88 | 0.0098 | F(1, 24) = 46.85 | < 0.0001 | F(1, 24) = 4.51 | 0.0442 |
| Fig. S1 D | *Dbp* mRNA | Time | Genotype | F(1, 24) = 8.31 | 0.0082 | F(1, 24) = 97.34 | < 0.0001 | F(1, 24) = 4.90 | 0.0367 |
| Fig. S1 D | *Rev-erbα* mRNA | Time | Genotype | F(1, 24) = 7.67 | 0.0106 | F(1, 24) = 57.71 | < 0.0001 | F(1, 24) = 5.67 | 0.0255 |
| Fig. S1 E | Urine volume | Time | Genotype | F(1, 24) = 4.79 | 0.0386 | F(1, 24) = 6.81 | 0.0153 | F(1, 24) = 9.78 | 0.0046 |
| Fig. S1 E | Urinary K excretion | Time | Genotype | F(1, 26) = 3.37 | 0.0779 | F(1, 26) = 11.84 | 0.0020 | F(1, 26) = 3.11 | 0.0896 |
| Fig. S1 E | Urinary Na excretion | Time | Genotype | F(1, 26) = 1.22 | 0.2804 | F(1, 26) = 2.44 | 0.1301 | F(1, 26) = 1.82 | 0.1885 |
| Fig. S1 F | Locomotor activity | Time | Genotype | F(1, 20) = 0.56 | 0.4649 | F(1, 20) = 4.00 | 0.0593 | F(1, 20) = 2.06 | 0.1672 |
| Fig. S1 F | Food intake | Time | Genotype | F(1, 20) = 3.55 | 0.0742 | F(1, 20) = 10.89 | 0.0036 | F(1, 20) = 0.22 | 0.6471 |
| Fig. S1 F | Water intake | Time | Genotype | F(1, 20) = 2.81 | 0.1093 | F(1, 20) = 46.81 | < 0.0001 | F(1, 20) = 2.25 | 0.1496 |
| Fig. S1 G | Na+ balance | Time | Genotype | F(1, 20) = 3.28 | 0.0851 | F(1, 20) = 10.21 | 0.0045 | F(1, 20) = 0.21 | 0.6508 |
| Fig. S1 G | K+ balance | Time | Genotype | F(1, 20) = 3.10 | 0.0935 | F(1, 20) = 9.24 | 0.0065 | F(1, 20) = 0.18 | 0.6720 |
